# NLRP3 protects alveolar barrier integrity by an inflammasome-independent increase of epithelial cell adherence

**DOI:** 10.1038/srep30943

**Published:** 2016-08-01

**Authors:** Elena Kostadinova, Catherine Chaput, Birgitt Gutbier, Juliane Lippmann, Leif E. Sander, Timothy J. Mitchell, Norbert Suttorp, Martin Witzenrath, Bastian Opitz

**Affiliations:** 1 Department of Internal Medicine/Infectious Diseases and Pulmonary Medicine, Charité - Universitätsmedizin Berlin, Augustenburger Platz 1, 13353 Berlin, Germany; 2 Institute of Microbiology and Infection, College of Medical and Dental Sciences, University of Birmingham, Birmingham, B15-2TT, UK

## Abstract

Bacterial pneumonia is a major cause of acute lung injury and acute respiratory distress syndrome, characterized by alveolar barrier disruption. NLRP3 is best known for its ability to form inflammasomes and to regulate IL-1β and IL-18 production in myeloid cells. Here we show that NLRP3 protects the integrity of the alveolar barrier in a mouse model of *Streptococcus pneumoniae*-induced pneumonia, and *ex vivo* upon treatment of isolated perfused and ventilated lungs with the purified bacterial toxin, pneumolysin. We reveal that the preserving effect of NLRP3 on the lung barrier is independent of inflammasomes, IL-1β and IL-18. NLRP3 improves the integrity of alveolar epithelial cell monolayers by enhancing cellular adherence. Collectively, our study uncovers a novel function of NLRP3 by demonstrating that it protects epithelial barrier function independently of inflammasomes.

Bacterial and viral pneumonias are the most common causes of acute lung injury (ALI) and acute respiratory distress syndrome (ARDS)^1^,^2^. Central to both conditions is an increased leakage of the alveolar epithelial and endothelial barriers, and an uncontrolled activation of inflammation as well as coagulation pathways^1^,^2^,^3^. The impairment of the alveolar barrier function can be caused by the inflammatory host response leading to recruitment of neutrophils to the alveoli and by microbial factors such as bacterial toxins. Whereas the molecular mechanisms of lung endothelial stabilization are increasingly well understood, less is known about the pathways that protect the epithelial barrier in the lung^1^.

*Streptococcus pneumoniae* is the major cause of community-acquired pneumonia, which can deteriorate to ALI and ARDS^4^,^5^. One of the most important pneumococcal virulence factors is the toxin pneumolysin (PLY)^6^,^7^. PLY is a member of the cholesterol-dependent cytolysins expressed by various Gram-positive bacteria. PLY of most pneumococci binds to cholesterol-containing membranes, forms large pores upon oligomerization, and thereby causes cell lysis. At sublytic concentrations, PLY has been additionally described to activate the complement system and to stimulate cytokine production in monocytes and macrophages^6^,^7^. Previous studies identified PLY as critical mediator of lung barrier disruption during pneumococcal pneumonia^8^,^9^,^10^,^11^. This detrimental effect is likely dependent on direct effects of PLY on the alveolar epithelial and/or endothelial cells, and may result in alveolar flooding and haemorrhage during the early stage of pneumococcal pneumonia^11^.

The NOD-like receptor molecule NLRP3 is best known for its ability to form inflammasomes. Canonical inflammasomes are macromolecular complexes consisting of a receptor molecule belonging to the NOD-like receptor (such as NLRP3) or the PYHIN protein families (e.g. AIM2), the adapter molecule ASC and caspase-1^12^,^13^. Inflammasomes regulate the production of IL-1β and IL-18 by a caspase-1-dependent processing of the cytokine pro-forms into mature cytokines, and induce a caspase-1-dependent inflammatory cell death called pyroptosis^12^. The NLRP3 inflammasome is activated in response to many pathogen-derived, environmental, and endogenous host molecules^14^. We and others recently showed that the NLRP3 inflammasome senses PLY activity upon pneumococcal infection, and mediates IL-1β and IL-18 production by macrophages and dendritic cells^15^,^16^,^17^,^18^,^19^. Unlike NLRP3 in myeloid cells, little is known about the function of this molecule in the epithelium, although several reports indicated that NLRP3 is expressed in different types of epithelial cells^20^,^21^,^22^.

In the present study, we employ different *in vivo, ex vivo*, and *in vitro* models to examine the effect of NLRP3 on the lung barrier integrity during pneumococcal infection and response to PLY. Surprisingly, we found that NLRP3 protects the alveolar wall by preventing detachment of epithelial cells. These lung barrier stabilizing effect of NLRP3 was independent of inflammasomes, IL-1β and IL-18. Our study thus uncovers a hitherto unknown function of NLRP3, which might have implications for the development of barrier stabilizing therapeutic strategies.

## Results

### NLRP3 protects the lung barrier during pneumococcal infection and treatment with PLY

**Figure 1:**
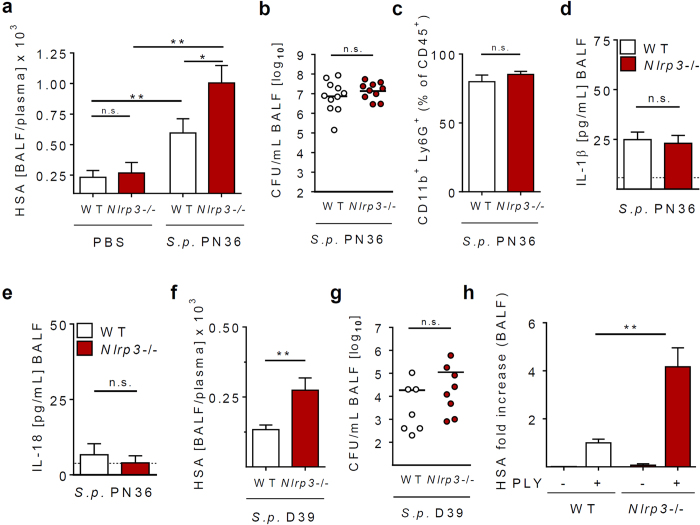
NLRP3 protects the alveolar barrier during pneumococcal pneumonia and PLY treatment. (**a**–**e**) 8–10 week old female mice were intranasally infected with 5 × 10^6^ *S. pneumoniae* PN36 for 24 h. (**a**) Lung permeability was quantified by measuring the HSA BALF/serum ratio after i.v. injection of HSA. Bacterial loads in BALF (**b**), neutrophil infiltration (**c**), as well as IL-1β (d) and IL-18 production (**e**) were measured 24 h post infection. (**f**,**g**) 8–10 week old female mice were intranasally infected with 5 × 10^6^ *S. pneumoniae* D39 for 24 h and lung permeability was quantified by measuring the HSA BALF/serum ratio in BALF after i.v. injection of HSA (**f**). Bacterial loads 24 h p.i were measured in BALF (****g****). (**h**) Isolated perfused and ventilated lungs from WT and *Nlrp3*−/− mice were intratracheally treated with PBS or PLY (0,2 μg/ml), and lung permeability was determined 30 min after stimulation by measuring leakage of HSA from the perfusion system to the broncho-alveolar space. Values are given as mean ± SEM; n = 7–11. *p < 0.05, **p < 0.01, n.s. = not significant.

In order to examine the effect of NLRP3 on lung barrier function during pneumonia, mice were intranasally infected for 24 h with a *S. pneumoniae* serotype 3 strain (PN36). Human serum albumin (HSA) was intravenously administered to measure the leakage of HSA from the circulation into the alveolar space at 24 hrs post infection (p.i.). In confirmation of previous studies^18^,^23^, we found that pneumococcal pneumonia increases alveolar-capillary barrier leakage as indicated by enhanced levels of HSA in bronchoalveolar lavage fluid (BALF) compared to uninfected mice (Fig. 1a). Next we compared WT and NLRP3-deficient animals, and found an increased *S. pneumoniae*-induced barrier dysfunction in *Nlrp3*−/− compared to WT animals at 24 h post infection (Fig. 1a). Neutrophil recruitment into the alveolar space, bacterial loads in the lung, as well as IL-1β and IL-18 production were not significantly affected by NLRP3 at this early time point (Fig. 1b–e). This unaltered cytokine production in *Nlrp3*−/− mice most likely reflects the involvement of additional inflammasomes (such as the AIM2 inflammasome) in controlling cytokine production^15^,^24^. *Nlrp3*−/− animals also showed enhanced lung barrier leakage (but not increased bacterial loads or altered cytokine production) after infection with a different pneumococcal strain (serotype 2 strain D39) in comparison to WT mice (Fig. 1f,g, and supplementary Fig. S1).

Given that PLY is critical for lung barrier dysfunction during pneumococcal pneumonia^8^,^9^,^10^,^11^, we next examined the effect of NLRP3 on the PLY-induced lung barrier dysfunction. Therefore, we used an *ex vivo* model which allowed for a more defined examination of NLRP3-mediated effects on barrier integrity, without the confounding influence of different host defence capacities in WT and *Nlrp3*−/− animals during pneumonia^4^. Isolated perfused and ventilated mouse lungs (IPMLs) were intravenously injected with HSA and challenged with PLY for 30 min to measure barrier integrity in analogy to the *in vivo* model (supplementary Fig. S2). Similarly to our *in vivo* results, we found that lungs of *Nlrp3*−/− animals exhibited increased HSA leakage compared to WT organs (Fig. 1g). These data demonstrate alveolar barrier stabilizing effects of NLRP3 during bacterial pneumonia and PLY treatment.

### NLRP3 enhances lung epithelial barrier function

**Figure 2:**
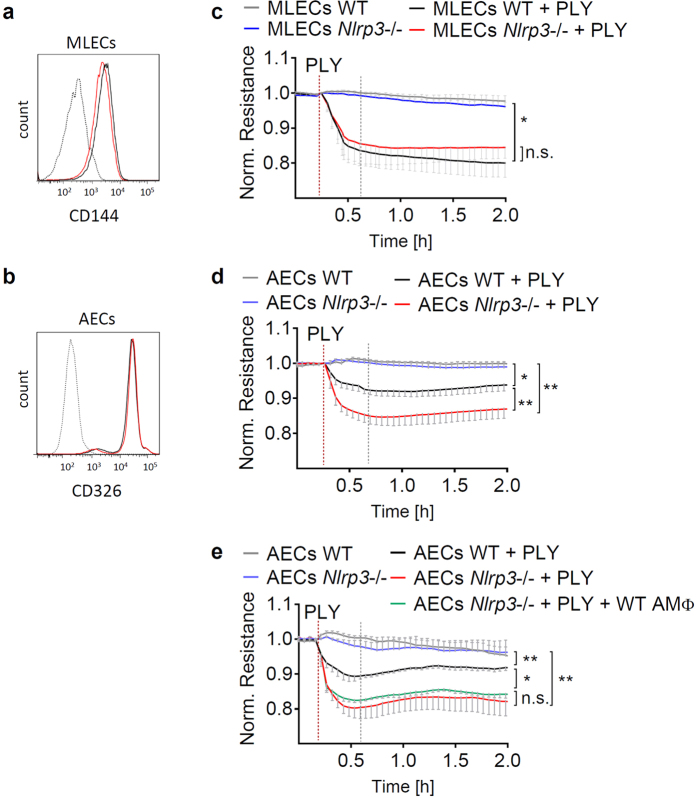
NLRP3 enhances resistance of alveolar epithelial cell monolayers against PLY-dependent interruption. MLECs and AECs from WT (black line) and *Nlrp3*−/− (red line) mice were isolated and their purity was determined by flow cytometry (**a**,**b**). MLECs (**c**) and AECs (**d**), as well as AECs co-cultured with alveolar macrophages (AMΦ) (**e**) were grown on ECIS electrodes. The cell monolayers were treated with 1 μg/mL PLY and resistance was measured continuously for 2 h by ECIS. Values represent a mean of at least 4 independent experiments performed in duplicates and are given as mean ± SEM. Statistical significance of the resistance drop was measured 30 min after PLY stimulation (indicated by grey dotted line). *p < 0.05, **p < 0.01, n.s. = not significant.

The alveolar barrier is a delicate structure measuring less than 1 μm in diameter and is composed of an inner endothelial and an outer epithelial cell layer, separated by the basal membrane. The integrity of the alveolar barrier is absolutely vital to maintain sufficient gas exchange and thus organ function. In order to shed light on the NLRP3-mediated barrier protective effects, we examined the resistance, as a marker of “tightness” of monolayers of epithelial and endothelial cells. Primary murine lung endothelial cells (MLECs) and alveolar epithelial cells (AECs) from WT and *Nlrp3*−/− animals were isolated (Fig. 2a,b) and grown on microelectrodes connected to an electrical cell-substrate impedance system (ECIS)^10^,^23^. Upon exposure to PLY, transcellular electrical resistance values from each microelectrode was continuously monitored and normalized to baseline resistance. As expected, PLY treatment led to a drop of the resistance indicating endothelial and epithelial barrier dysfunction (Fig. 2c,d). Notably, NLRP3-deficient AECs showed enhanced barrier dysfunction in response to PLY treatment, whereas lack of NLRP3 did not influence the MLEC barrier function. The purity of isolated AECs was greater than 95%, rendering responsibility of contaminating macrophages for the protective effect of NLRP3 unlikely. Moreover, addition of WT alveolar macrophages (AMΦ) to *Nlrp3*−/− AEC monolayers did not affect barrier resistance (Fig. 2e). Collectively, these data suggest that intraepithelial NLRP3 protects the lung epithelial barrier function.

### The protective effect of NLRP3 on the alveolar barrier is independent of inflammasomes, IL-1β and IL-18

**Figure 3:**
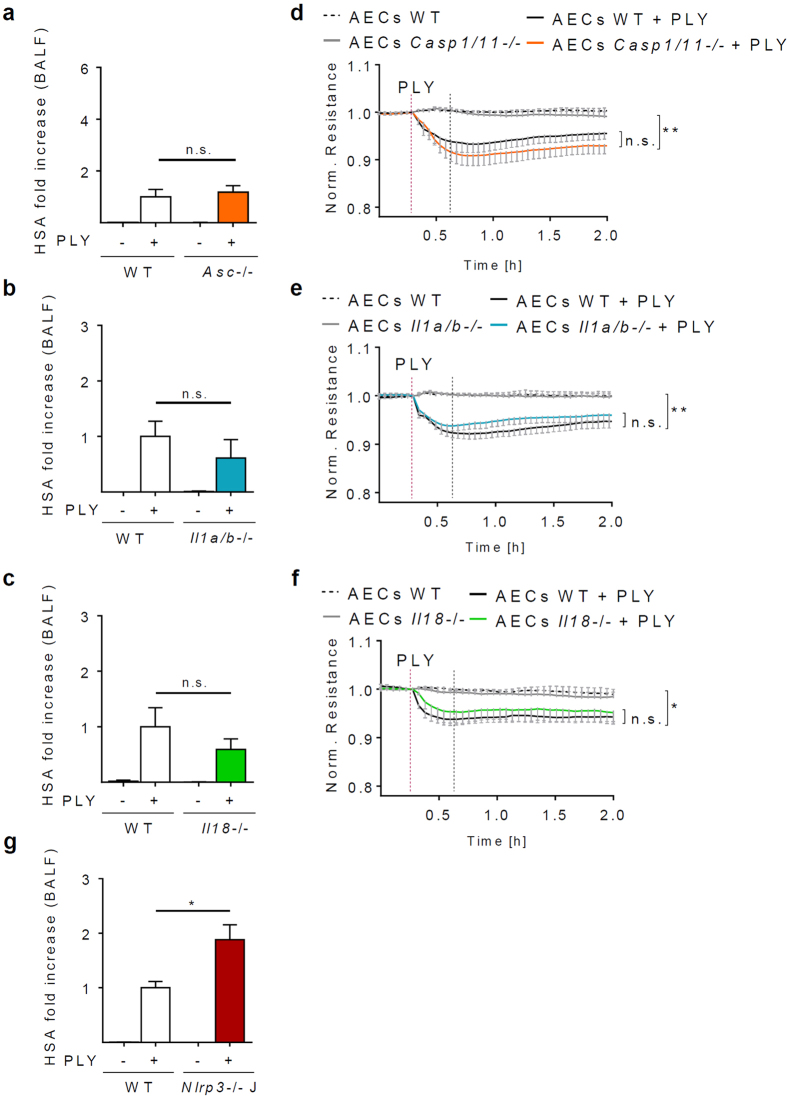
The protective effect of NLRP3 for the alveolar permeability is independent of inflammasomes, IL-1β and IL-18. (**a**–**c**). Permeability of isolated perfused and ventilated lungs from WT, *Asc*−/−, *Il1a/b*−/− and *Il18*−/− was measured by HSA ELISA. (**d**–**f**) AECs isolated from WT, *Casp1/11*−/−, *Il1a/b*−/− and *Il18*−/− mice and grown on gold electrodes were treated with 1 μg/ml PLY and resistance was measured continuously for 2 hours. (**g**) Isolated perfused and ventilated lungs from WT and *Nlrp3*−/−J (Jax^®^ mice) mice were intratracheally treated with PBS or PLY (0.2 μg/lung), and lung permeability was determined 30 min after stimulation by measuring leakage of HSA from the perfusion system to the broncho-alveolar space. Values represent a mean of 4–12 independent experiments and are given as mean ± SEM. Statistical significance of the resistance drop was measured 30 min after PLY stimulation (indicated by grey dotted line). *p < 0.05, **p < 0.01, n.s. = not significant.

The NLRP3 is best known for its role in forming inflammasomes and in controlling IL-1β as well as IL-18 production^12^,^14^, and IL-18 has been implicated in protecting the intestinal barrier^25^,^26^. We therefore tested if (additional) inflammasome components, IL-18 or IL-1β are involved in lung barrier protection by NLRP3. Surprisingly, we found that PLY treatment led to a comparable barrier dysfunction in lungs from WT, *Asc*−/−, *Il1b*−/− and *Il18*−/− mice *ex vivo* (Fig. 3a–c). Similarly, deficiency of Caspase-1/11, IL-1 or IL-18 had no effect on the resistance of AEC monolayers as measured by ECIS (Fig. 3d–f). In order to exclude the possibility that the NLRP3-specific effects on the lung barrier are caused by a secondary mutation in the mouse strain used, we examined lungs of an independently generated NLRP3-deficient mouse strain (obtained from The Jackson laboratory) in our *ex vivo* IPML model. Similarly to our results shown in Fig. 1g, we found the PLY-induced barrier dysfunction was enhanced in Jax^®^ *Nlrp3*−/− mice (*Nlrp3*−/−J) compared to WT controls (Fig. 3h). Collectively, these data demonstrate that NLRP3 protects the lung epithelial barrier independently of inflammasomes, IL-1β and IL-18.

### NLRP3 prevents detachment of lung epithelial cells from fibronectin surfaces

**Figure 4:**
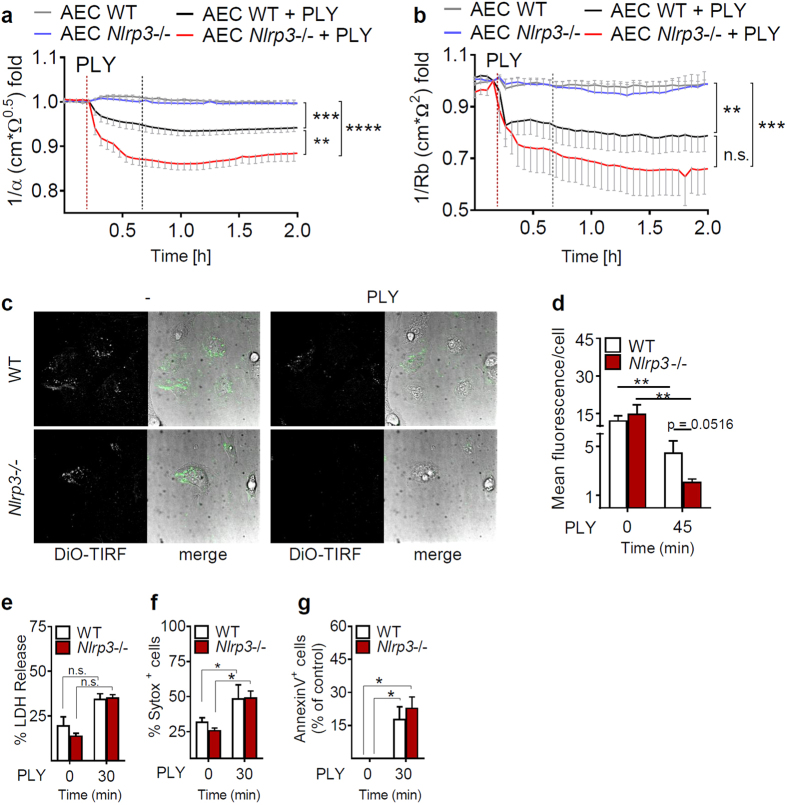
NLRP3 enhances attachment of alveolar epithelial cells. Attachment (**a**) as well as junctional stability (**b**) upon PLY treatment of WT and *Nlrp3*−/− AECs was determined by measuring the alpha (α) and Rb parameters using ECIS. Values represent a mean of ten independent experiments performed in duplicates. (**c**,**d**) AECs were stained with the membrane dye DiO and subsequently subjected to TIRFM life imaging. Pictures were taken at 30 min after adding medium without (−) and 45 min after changing media containing PLY (1 μg/ml). (**c**) Representative DiO-TIRF and DIC images are shown. (**d**) Particle analysis was performed from four fields from two independent experiments and values were normalized to cell numbers and average control values. (**e**,**f**,**g**) Cell death of WT and *Nlrp3*−/− AEC treated for 30 min with PLY (1 μg/ml) was measured by LDH release (**e**), Sytox staining (**f**) and AnnexinV staining (**g**). (**e**,**f**,**g**) Data represent the mean of three independent experiments. All data are given as mean ± SEM *p < 0.05, **p < 0.01, ***p < 0.001, ****p < 0.0001, n.s. = not significant.

Using a mathematical tool of the ECIS software, the resistance of cell monolayers measured by ECIS can be divided into two parameters. α is the electrical current flow under the cells, representing the adhesion of the cell layer on the surface. Rb is the current flow between cells and represents the permeability of the cell barrier^27^,^28^,^29^,^30^. To determine the effect of NLRP3 on the adhesion and permeability of alveolar epithelial cell layers, we treated WT and *Nlrp3*−/− cells with PLY and plotted the 1/α and 1/Rb over time. The epithelial cell adhesion was significantly decreased in PLY-treated *Nlrp3*−/− compared to WT cells (Fig. 4a), whereas the current flow between cells was not significantly different between WT and *Nlrp3*−/− cells (Fig. 4b). In order to confirm our results obtained from ECIS measurements showing diminished cellular adhesion in *Nlrp3*−/− cells, we performed live TIRF microscopy (TIRFM) using a lipophilic membrane dye. Fluorescence intensity correlates with areas of cellular adhesion within 200 nm distance from the glass bottom and can be quantified using a particle analysis tool. We observed loss of cellular adhesion upon addition of PLY in AECs, which seemed to further decrease by lack of NLRP3 (Fig. 4c,d). However, although we observed a moderate increase in apoptotic and/or necrotic epithelial cell death upon PLY treatment, cell death was not affected by NLRP3 deficiency (Fig. 4e–g). In summary, these results indicate that NLRP3 improves the attachment of AECs to fibronectin-containing matrices.

## Discussion

We demonstrate here that NLRP3 protects the alveolar barrier upon pneumococcal infection or PLY challenge. Previous studies showed that epithelial NLRP3 and NLRP6 inflammasomes protect the intestinal barrier integrity by stimulating an IL-18-dependent cell proliferation in the reconstitution phase after DSS-induced injury^25^,^26^,^31^. We initially hypothesised that it might protect the alveolar epithelium via a similar inflammasome- and IL-18-dependent mechanism. However, the compromised lung barrier function in *S. pneumoniae*-infected *Nlrp3*−/− compared to WT animals was not associated with decreased levels of IL-1β and IL-18 at the tested time point. Moreover, lack of ASC, IL-18 or IL-1β did not affect the barrier function in isolated perfused and ventilated lungs. Finally, AEC monolayers of *Casp1*−/−, *Il1b*−/− or *Il18*−/− mice did not demonstrate enhanced barrier dysfunction upon PLY treatment. Although we cannot exclude the possibility of additional inflammasome- and/or IL-18-dependent barrier preserving mechanisms in the lung at the healing stage, our results clearly indicate that NLRP3 is able to protect the alveolar epithelium in an inflammasome-, IL-18- and IL-1β-independent fashion during the acute stage of injury.

There have been previous reports indicating the involvement of inflammasome components in cytokine-independent functions in the epithelium. NLRP3, for example, has been shown to contribute to renal ischemia-reperfusion injury independently of ASC, IL-1β and IL-18^32^. Moreover, inflammasome-independent NLRP3 was shown to facilitate the epithelial-mesenchymal transition of kidney epithelial cells by enhancing TGF-β signaling and R-Smad activation^21^.

In our study, measurement of the current flow under AEC monolayers as well as TIRF microscopy demonstrated increased detachment of *Nlrp3*−/− AECs compared to WT cells upon PLY treatment, which was not associated with enhanced cell death. These results suggest that NLRP3 increases AEC attachment to fibronectin-containing matrices, such as the basal membrane. The mechanism of how NLRP3 affects the adherence of AECs and whether this is mediated by type I or II AECs is unclear at this stage and should be elucidated in future studies.

Interestingly, NLR molecules might be able to regulate epithelial attachment and detachment in different ways. Hardt *et al*. recently showed that NAIP5 controls expulsion of infected intestinal epithelial cells from monolayers independently of IL-1β and IL-18^33^. Future studies should further explore how specific NLRs influence cell adhesion as well as junctional molecules on epithelial cells in different organs.

In conclusion, our study reveals a novel function of NLRP3 in lung barrier protection independently of inflammasomes, IL-1β and IL-18. In the future, therapeutic stimulation of NLRP3 might be useful to stabilize the lung barrier function during pneumonia.

## Methods

### Bacterial strains

*S. pneumoniae* serotype (ST)3 strain PN36 (NCTC7978) and ST2 strain D39 strain were used. Bacteria were grown in THY media at 37 °C and 5% CO_2_ until reaching a logarithmic growth phase. Pneumolysin (PLY) was produced and purified as previously described^34^.

### Murine pneumonia model

Animal procedures were approved by local authority (LAGeSo Berlin, Charite Berlin) and the methods were carried out in accordance with the approved guideline. 8–10 week female mice were anesthetized by i.p. ketamine (1.6 mg) and xylazine (0.5 mg) and transnasally infected with 5 × 10^6^ CFU *S. pneumoniae* PN36 or D39 for 24 h^18^,^23^,^35^. To estimate lung microvascular leakage, human serum albumin (HSA) was infused intravenously 1 h before broncheo-alveolar lavage (BAL). HSA concentration in BAL fluid (BALF) and serum was measured by ELISA, and the HSA BALF/serum ratio was calculated

### Determination of bacterial load and cell recruitment

Bacterial loads were determined in BALF and blood. Serial dilutions of samples were plated on blood agar and colony forming units (CFU) were counted. To test for cell recruitment, cells were enriched by spinning the BALF (400 g 10 min RT), incubated with blocking antibody (anti-CD16/32, Biolegend) and stained using CD11c-FITC (N418, Biolegend), SiglecF-PE (E50-2440, BD bioscience), Ly6C-PerCp (HK1.4, Biolegend), F4/80-APC (BM8, Biolegend), CD45-Alexa700 (30-F11, Biolegend), Ly6G-BV421 (1A8, Biolegend) and CD11b-BV510 (M1/70, Biolegend).

### ELISA

Concentrations of murine IL-1β in BALF or in cell-free supernatants were quantified by commercially available sandwich ELISA kit (eBioscience, Frankfurt, Germany; R&D, Minneapolis, USA). Murine IL-18 was measured by specific ELISA (R&D Systems, UK, and MBL, Japan).

### Isolated perfused and ventilated mouse lung (IPML)

Lungs of wild-type (WT), *Nlrp3*−/−36, *Nlrp3*−/−J (The Jackson Laboratory, Stock No: 021302) and *Asc*−/−37 mice were prepared according to the experimental setup described previously^23^. In short, mice were anesthetized and placed in a 37 °C chamber. After laparotomy, sternotomy, and cannulation (left atrium, pulmonary artery), lungs were perfused with electrolyte solution (Serag-Wiessner, Germany) supplemented with sodium bicarbonate. The chamber was closed and lungs were ventilated and perfused for 20 min to establish baseline conditions. 10 min prior to PLY application, HSA was added to the perfusate. For PLY-stimulation, the chamber was opened and lungs were treated intratracheally with 0,2 μg toxin/lung. At 30 min perfusion/ventilation with closed chamber albumin concentration was measured in the BAL fluid after the experiment using an ELISA (Bethyl, US).

### Isolation of primary cells

Primary cells were isolated from WT, *Nlrp3*^−/−^, *casp1*^−/−^, *Il1a/b*^−/−^ and *Il18*^−/−^ animals on a C57Bl/6J background. For isolation of alveolar epithelial cells (AECs), lung homogenates were prepared and leukocytes and endothelial cells were depleted from the cell suspension by incubation with biotinylated rat anti-mouse CD45, CD16/32 and CD31 (BD Pharmingen, Heidelberg, Germany) followed by magnetic separation. AECs were cultured for 4 days in DMEM medium supplemented with 25 mM HEPES, 1% Glutamine, 1% Penicilin/Streptomycin and 10% FCS. Murine lung endothelial cells (MLECs) were isolated from lung homogenates by positive magnetic selection using biotinylated rat anti-mouse CD144 (BD Pharmingen). MLECs were grown in Endothelial Cell Growth Medium MV2 (Promocell) supplemented with 1% Penicilin/Streptomycin and 15% FCS. Isolated cells were washed and stained with CD326-BV421 (G8.8, Biolegend) for epithelial cells and CD144-Alex647 (BV13, Biolegend) for endothelial cells, respectively. For cell culture, plates were coated with fibronectin (Sigma-Aldrich, USA). Alveolar macrophages (AMΦ) were obtained by BALF from mouse lungs. For co-culture experiments, AMΦ were isolated 1 day before the experiment and were added to AEC cultures in a ratio of 1:20.

### Electric Cell Substrate Impedance Sensing (ECIS)

ECIS measurements of epithelial and endothelial cell monolayers were performed as described previously for endothelial cells^23^. AECs and microvascular endothelial cells (MLECs) were seeded on Fibronectin-coated 8W10E+ electrodes (Applied Biophysics) and grown to confluence. The cells were treated with 1 μg/ml PLY (for AECs) and 0.5 μg/ml PLY (for MLECs). Monolayer resistance was measured at 37 °C and 5% CO_2_ using a 1600R ECIS system (Applied Biophysics) at 4000 Hz. ECIS software (v1.2.50.0 PC, Applied Biophysics) was used to calculate the alpha (α) and the Rb parameter. Resistance data at 30 min after PLY treatment were statistically analyzed by Student’s t-test (two-tailed, unpaired).

### Flow Cytometry

All the FACS data were acquired on FACS CantoII (Becton Dickinson, Franklin Lakes, NJ, USA). Gating strategy was processed after exclusion of the doublets. Data were analysed using the data analysis software, FlowJo (Ashland, OR, USA).

### Total internal reflection fluorescence microscopy (TIRFM)

For TIRFM, AECs were grown on fibronectin-coated μ-slide 8 well glass bottom slides (Ibidi) for 4 days and stained with 5 μl/ml Vybrant DiO cell labeling solution (Thermofisher) for 10 min at 37 °C. Life imaging was performed in the imaging facility of the Molecular Biophysics group, HU Berlin on a Confocal Laser Scanning Microscope (Olympus FV-1000MPE) equipped with a TIRFM upgrade at 37 °C and 5% CO_2_ using a 63x oil objective and a cooled CCD camera. Images were taken at indicated time points and analysed using FijI. Particle analysis was performed from binary images after thresholding (method Isodata). Total intensity of attachment from all detected particles was measured.

### Cell death analysis

Cell death of PLY-treated AECs (30 min, 1 μg/ml) was determined by the spontaneous release of lactate dehydrogenase (LDH) using the CytoTox 96 Assay (Promega). LDH assay was performed according to the manufacturer’s instructions. Moreover, to confirm the cell death data, SYTOX Orange Nucleic Acid stain (Molecular probes, Thermofisher Scientific) was used. AECs were pre-incubated SYTOX Orange for 10 min (dilution 1:10 000) and the increase in fluorescence intensity upon PLY treatment was measured with FilterMax F3 Multi-Mode Microplate Reader (Molecular Devices, USA). Additionally, AnnexinV-APC (Biolegend) was used following the instruction of manufacturer. The data were standardized by subtracting the percentage of PLY-treated AnnexinV+ cells from the percentage of AnnexinV+ cells in non-treated samples.

### Data analysis

Data are expressed as mean ± SEM. For comparison of two groups, Mann-Whitney U Test was used when the data values were not normally distributed. Student t-test was used for the particle analysis of TIRFM data. Data analysis was performed using the Prism software (GraphPad Software, La Jolla, CA). For all statistical analysis, p values < 0.05 were considered significant with *p < 0.05,**p < 0.01, ***p < 0.001, ****p < 0.0001.

## Additional Information

**How to cite this article**: Kostadinova, E. *et al*. NLRP3 protects alveolar barrier integrity by an inflammasome-independent increase of epithelial cell adherence. *Sci. Rep.* **6**, 30943; doi: 10.1038/srep30943 (2016).

## Supplementary Material

Supplementary Information
