# Supplementary material for: NLRP3 protects alveolar barrier integrity by an inflammasome-independent increase of epithelial cell adherence
Source: Sci Rep. 2016 Aug 1;6:30943. doi: 10.1038/srep30943 (PMC4967923; doi:10.1038/srep30943)
Supplement: Supplementary Information [file srep30943-s1.pdf]

Supplementary Information

NLRP3 protects alveolar barrier integrity by an inflammasome-independent increase of epithelial cell adherence

Elena Kostadinova[1,3], Catherine Chaput[1,3], Birgitt Gutbier[1,3], Juliane Lippmann[1], Leif E. Sander[1], Timothy J. Mitchell[2], Norbert Suttorp[1], Martin Witzenrath[1], Bastian Opitz[1,*]

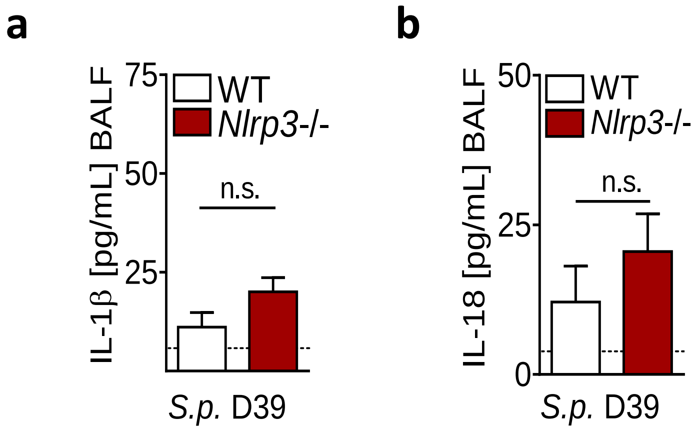

Supplementary Fig. S1: Cytokine production in WT and *Nlrp3-/-* mice upon *S. pneumoniae* D39 infection. 8-10 week old female mice were intranasally infected with 5×10^6 *S. pneumoniae* D39 for 24 h and cytokine production was measured in BALF. n = 5-7, data are given as mean ± SEM.

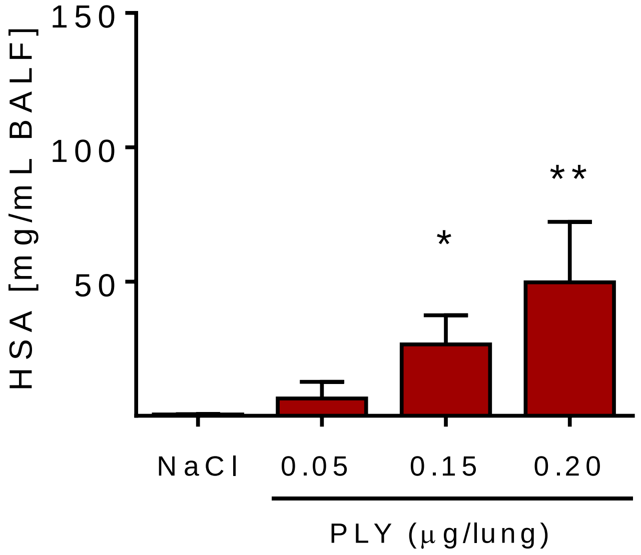

Supplementary Fig. S2: Dose-dependent increase in lung permeability upon PLY treatment in isolated perfused and ventilated lungs (IPML). IPML from WT mice were intratracheally treated with a range of PLY doses and lung permeability was determined 30 min after stimulation by measuring leakage of HSA from the perfusion system to the broncho-alveolar space. n = 5-7; data are given as mean ± SEM. *p < 0.05, **p < 0.01, n.s. = not significant.
